# Design Analysis and Isotropic Optimization for Miniature Capacitive Force/Torque Sensor

**DOI:** 10.3390/s25030940

**Published:** 2025-02-04

**Authors:** Seung Yeon Lee, Jae Yoon Sim, Yong Bum Kim, Dongyeop Seok, Jaeyoon Shim, Hyouk Ryeol Choi

**Affiliations:** 1 Department of Mechanical Engineering, Sungkyunkwan University, Suwon 16419, Republic of Korea; sylee6051@skku.edu (S.Y.L.); sjaeyoon@skku.edu (J.Y.S.); wodbs1118@g.skku.edu (J.S.); 2 Aidin Robotics Inc., Anyang 14055, Republic of Korea; ybkim@aidinrobotics.co.kr (Y.B.K.); dy.seok@aidinrobotics.co.kr (D.S.)

**Keywords:** robotic sensing, capacitive sensor, six-axis F/T sensor, optimal design and analysis

## Abstract

A capacitive six-axis force/torque (F/T) sensor has favorable characteristics for miniature design. However, when designing small-sized force/torque sensors, anisotropy among the six axes can lead to uneven sensitivity across each axis. This is due to increased crosstalk errors, which degrade sensor performance. To design a miniature six-axis force/torque sensor, it is essential to analyze the isotropic relationships between the six-axis forces/torques and the capacitance change to reduce crosstalk errors. This paper presents a miniature capacitive six-axis F/T sensor optimized for isotropy. It also establishes a systematic method for designing sensing electrodes. The sensor’s deformable structure is analyzed using Castigliano’s beam theory, and design parameters are optimized with isotropy analysis of the deformable part. The criteria are also presented, including selecting the electrode area and initial gap using linear equations derived from capacitance change analysis. The optimized miniature F/T sensor is calibrated using a neural network-based calibration method, and its accuracy errors are compared to a reference sensor. The design framework provides a foundation for future developments in miniature sensors.

## 1. Introduction

Sensing force and torque is crucial for interactions between robots and humans. A six-axis F/T sensor is an essential component that enables a robot to feel force and torque. The three-axis orthogonal force and torque information of a six-axis F/T sensor is necessary for advanced applications such as collaborative robots, force feedback teleoperation systems, grippers, and hands [1,2,3,4]. Therefore, it enables the establishment of reliable force control tasks for robotic solutions such as direct teaching, polishing, peg-in-hole tasks, and human–robot interaction (HRI).For this reason, embedding an F/T sensor in the fingertip enables the manipulation of an object and the execution of delicate tasks through force control, improving agility in robotic applications such as parallel jaw grippers and hands [5]. The fingertip is an interactive direct point of contact between a robot and an object. The fingertip sensor’s contact data enables the calculation of normal and tangential forces, which are subsequently utilized in the hand’s force feedback control system [6,7]. Related research has also been conducted on attaching force sensors not only to the fingertips but also to the main links of the finger mechanism to measure external contact force. In mechanisms similar to the human finger, composed of flexible fingertips and rigid links, integrating force sensors into the main links may be an effective method to calculate fingertip forces [8]. For stable grasping by grippers or hands, it is necessary to embed small-sized force/torque sensors in the jaws and fingertips where contact with objects occurs and also rigid links [9].

However, embedding a commercial six-axis F/T sensor in the fingertip has practical limitations such as the sensor’s size, weight, and high cost. Also, a sensitivity anisotropy problem occurs as the sensor size decreases [10]. In addition, the xy-axis sensitivity is too high compared with the z-axis force sensitivity. Despite the importance of sensitivity in the z-axis direction, there remains a capacitive anisotropic problem that needs optimization of the force/torque ratio [11]. Increasing anisotropy of the force/torque sensitivity ratio means an increasing coupling effect among the six axes [12,13]. It causes an increase in the sensors’ accuracy error. However, capacitive type F/T sensors still face challenges when optimizing deformable parts’ design parameters. Furthermore, even when designing an optimized isotropic deformable part, selecting the area and initial gap between electrodes in a capacitive force/torque sensor remains a crucial design factor. Although various design criteria exist for the deformable part, the design criteria for the sensing electrode that measures capacitance are still insufficient [14].

This paper presents a miniature capacitive-type six-axis F/T sensor via an optimal design framework using the condition number. The criteria for selecting the area of the sensing electrode and the initial gap between it and the ground are also presented. The relationship between the external six-axis F/T and the displacement of deformable parts is analyzed based on Castigliano’s beam theory. Furthermore, the relationship between displacement and capacitance change is formulated. Therefore, the optimal design parameters are determined using the condition number to achieve isotropic sensitivity for the six-axis F/T. The capacitance change can be calculated by analyzing the relationship between force/torque and the deformable part’s displacement. The deformable part refers to a structure that changes in response to the external force/torque, and the change amount means displacement. The fundamental principle of a capacitive-type force/torque sensor is to measure the distance change between the electrode and the ground, acting as a sensing electrode. This distance changes due to the displacement of the deformable part, which is the ground role. The changed distance corresponds to a capacitance change. In other words, the displacement of the deformable part indicates the capacitive sensor’s sensitivity. To achieve sensitivity isotropy, the point where the capacitance change becomes identical across all axes is identified. Using this identified point as a criteria point, a linear equation can be derived with the electrode area and initial gap as variables. Additionally, the area of the electrodes and the electrode gap are crucial design factors that determine the sensor’s sensitivity performance. Therefore, the optimal design parameters for the sensing electrode (area and electrode gap) should be selected based on the isotropic design parameters of the deformable part. Section 2 describes the mathematical analysis of the deformable structure using Castigliano’s beam theory. Section 3 introduces the process of finding the optimal design parameters using the condition number and criteria for designing the sensing electrodes. Finally, Section 4 discusses the performance analysis and evaluation of the developed sensor.

## 2. Analysis of the Deformable Part

**Figure 1:**
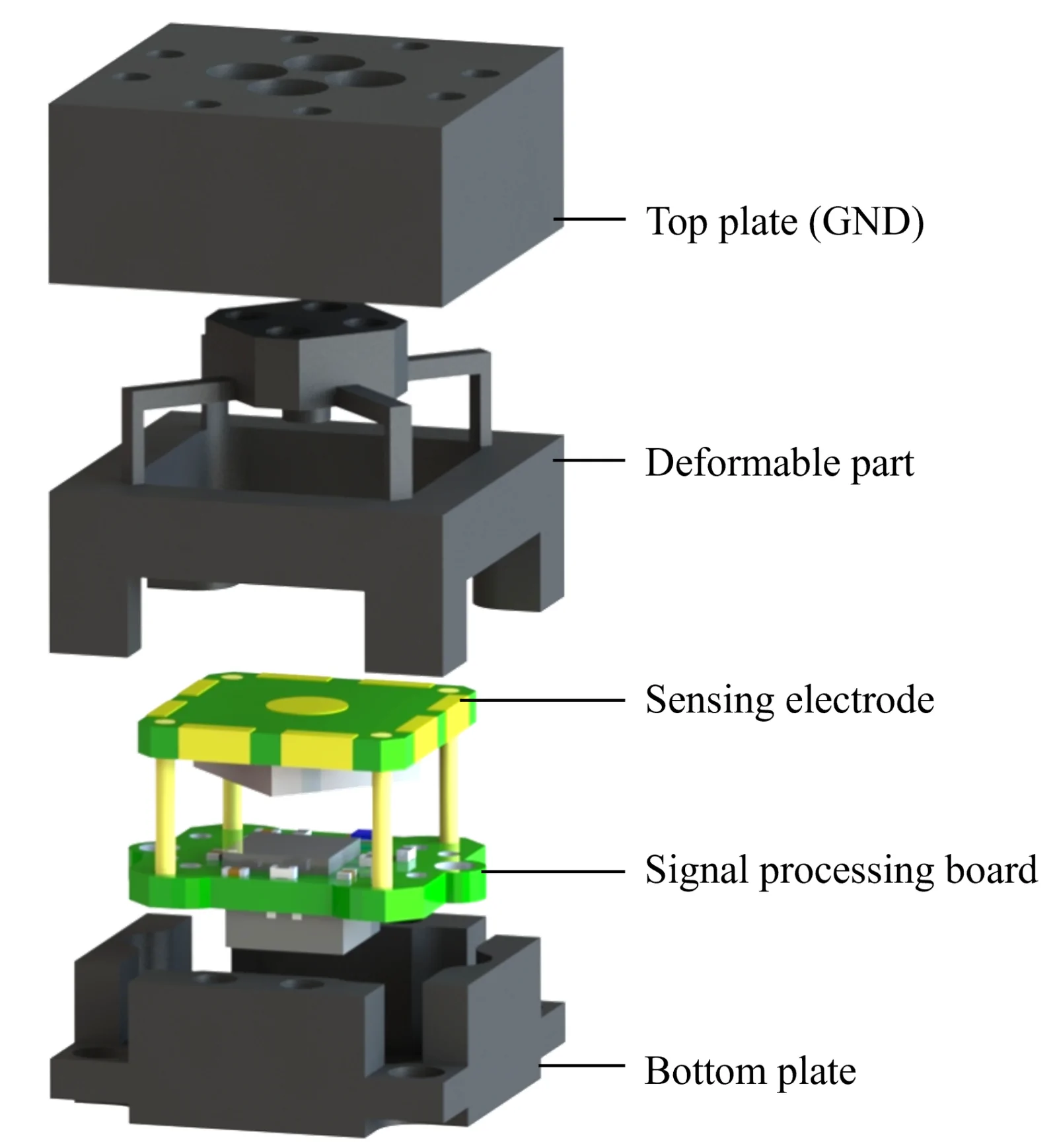
Exploded view of the developed capacitive six-axis F/T sensor.

A capacitive-type six-axis F/T sensor has some advantages: fabrication at an affordable cost, an all-in-one design without external devices like an amplifier, and ease of customization for robotic applications. As shown in Figure 1, the assembly components consist of five elements: the top plate, the deformable part, the sensing electrode, the signal processing board, and the bottom plate. The top plate serves as the ground (GND), and when the sensor is assembled, it creates an air gap between the GND and sensing electrode. When an external F/T is applied to the top plate, it causes displacement of the deformable part, which leads to a capacitance change.

### 2.1. Capacitive Sensor Sensing Principle

**Figure 2:**
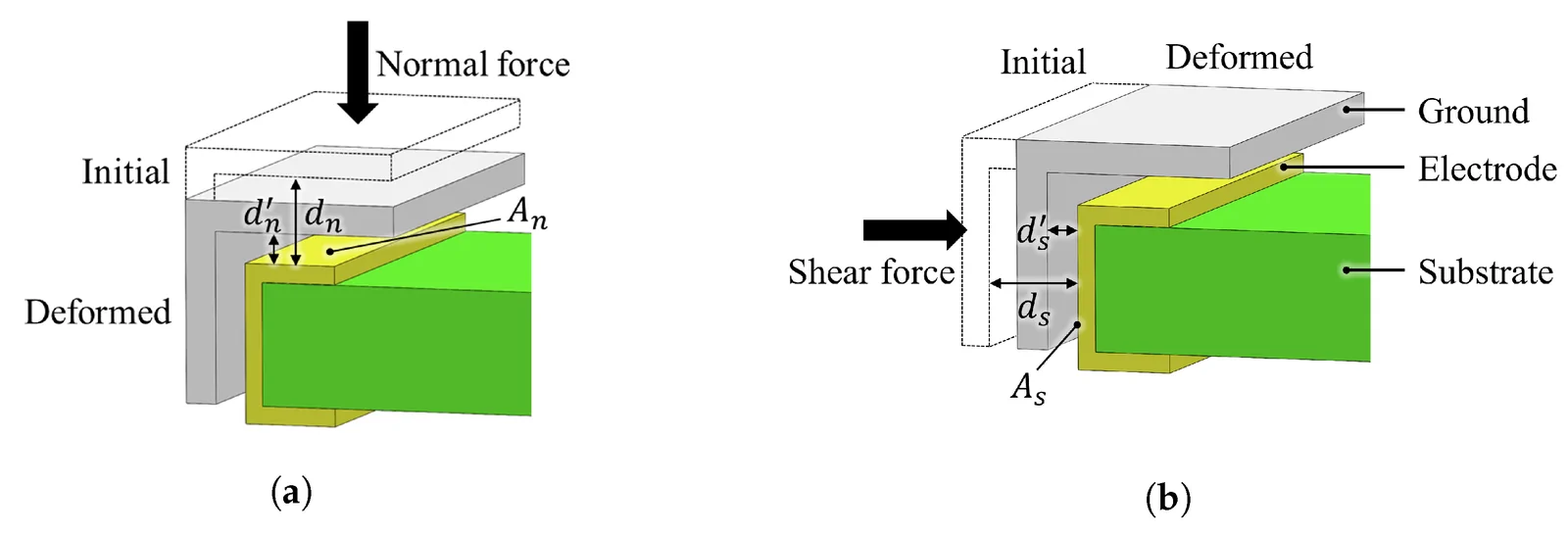
Sensing electrode. (**a**) Sensing electrode change due to normal force. (**b**) Sensing electrode change due to shear force.

As shown in Figure 2, the ground part and electrode are arranged parallel to each other to create an air gap for capacitance sensing. A capacitance field is formed in the air gap, and the ground part moves from its initial position to a deformed position due to the displacement caused by external forces. The ground part moves from $d_{n}$ to $d_{n}^{\prime }$ due to the normal force, and similarly, it moves from $d_{s}$ to $d_{s}^{\prime }$ due to the shear force. This position change indicates that the displacement and capacitance change in the normal and shear axes. Hence, the variation between *d* and $d^{\prime }$ caused by external forces indicates the sensitivity of the displacement and capacitance changes. To measure the six-axis F/T, it is necessary to acquire displacement data from at least six distinct directions and positions [5]. As illustrated in Figure 2a,b, the six-axis F/T is measured within the sensor by separating them into normal and shear forces, respectively [9]. By combining at least three normal forces and three shear forces, it is possible to extract the six-axis F/T. The initial capacitance value can be expressed as the initial electrode distance and area; this relationship can be written as

(1) $$\begin{array}{c} \begin{array}{c} C_{init}=\varepsilon_{0}\varepsilon_{r}\frac{A_{init}}{d_{init}} \end{array} \end{array}$$

where $C_{init}$ is the initial capacitance, and $A_{init}$ and $d_{init}$ represent the overlapping area and initial distance, respectively. $\varepsilon_{0}$ and $\varepsilon_{r}$ represent the free-space permittivity and the air’s relative dielectric constant, respectively. $\varepsilon_{0}$ and $\varepsilon_{r}$ are constant, and also, even when an external force is applied, the overlapped initial area $A_{init}$ between the ground part and the electrode remains constant. So, the capacitance change can be expressed as follows:

(2) $$\begin{array}{c} \begin{array}{c} ∆C=C_{init}-C_{def}=\varepsilon_{0}\varepsilon_{r}A\left(\frac{1}{d_{init}}-\frac{1}{d_{def}}\right) \end{array} \end{array}$$

where $C_{def}$ is the deformed position of the ground, $d_{def}$ is the deformed distance, and *A* is the overlap area. Furthermore, Equation (2) can be approximated using a Taylor series [15]. Using the third-order term of the Taylor series, ∆C can be calculated as follows:

(3) $$\begin{array}{c} \begin{array}{c} ∆d=d_{init}-d_{def} \end{array} \end{array}$$

(4) $$\begin{array}{c} \begin{array}{c} ∆C\approx \varepsilon_{0}\varepsilon_{r}A\left(-\frac{∆d}{d_{init}^{2}}+\frac{∆d^{2}}{d_{init}^{3}}-\frac{∆d^{3}}{d_{init}^{4}}\right) \end{array} \end{array}$$

where Equation (3) ∆d represents the displacement of the deformable part. Based on the fundamental Equation (4) of capacitance, the parameters of ∆d, $d_{init}$, and *A* significantly influence the performance of capacitive sensors. For example, even if the same ∆d occurs in different sensors, a sensor with a closer $d_{init}$ has higher sensitivity. Hence, displacement ∆d, initial distance $d_{init}$, and area *A* are key factors in sensor design.

Displacement ∆d, one of the crucial factors of sensor performance, is determined by the deformable part. The deformable part can take various forms, such as Y-shape, T-shape, and L-shape [16,17,18]. Among these, the L-shape structure has advantages such as ease of fabrication and a convenient arrangement of capacitive electrodes. It is a suitable structure for minimized capacitive F/T sensors. Despite having these advantages, the design becomes much more complex as the size of the deformable part is minimized. In addition, the anisotropy of the deformable part increases, further complicating the selection of design parameters. Although humans can determine design parameters through repetitive trial-and-error processes, this approach is highly time-consuming, and identifying the optimal design parameters remains challenging. For this reason, it is necessary to establish criteria for the design parameters by analyzing the mechanical model of the deformable part.

### 2.2. Analysis of Deformable Part

This section proposes a method for determining the criterion for minimized deformable parts, considering the six-axis isotropy of the L-shaped deformable part. One method for optimizing design parameters considering isotropy is using condition numbers. The condition number represents the sensitivity of a system to input variations and serves as a measure of isotropy. A condition number close to 1 is ideal and indicates excellent isotropy; it means a uniform system response to applied forces and torques across all axes [19,20]. Additionally, a deformable part with isotropy exhibits uniform sensitivity in all directions and minimizes coupling errors, ensuring independent and consistent responses to forces and torques applied along each axis. A stiffness or compliance matrix of a deformable part should be mathematically analyzed and defined. The compliance matrix is critical for defining the design criterion and is a basis for evaluating and optimizing its performance regarding isotropy and sensitivity.

**Figure 3:**
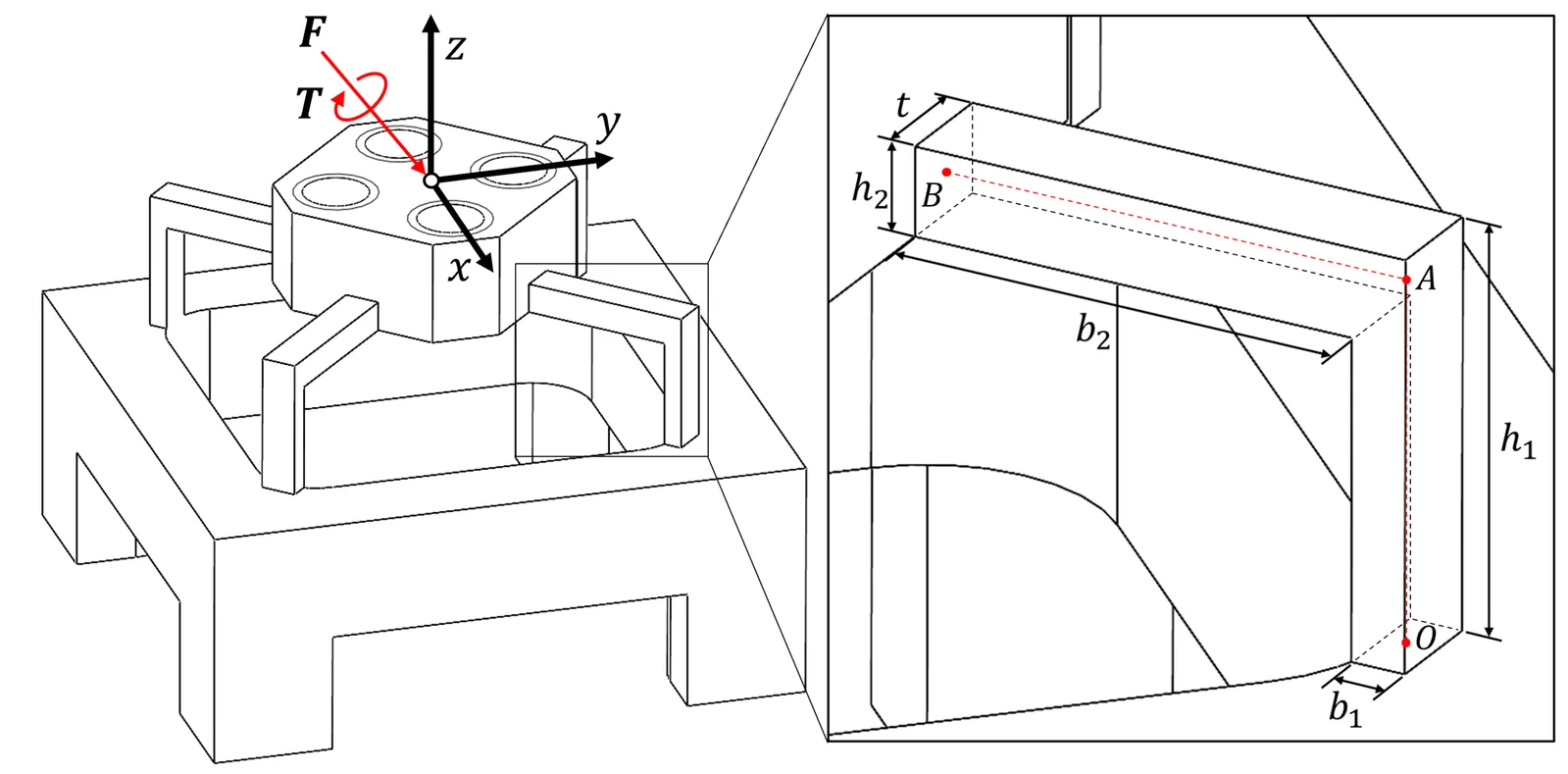
A mechanical model of the deformable part and L-shaped design parameters.

As shown in Figure 3, a mechanical model of the L-shape deformable part is established.

The deformable part is represented in a three-dimensional cartesian coordinate system, and F,T denotes the external force and torque. The mechanical model is based on the geometric characteristics and is divided into five design parameters $\left(b_{1},b_{2},h_{1},h_{2},t\right)$. By dividing the structure into nodes (e.g., *B*, *A*, and *O*), the model is parameterized to capture key design parameters. These parameters include geometric dimensions, such as arm lengths and thicknesses, that influence the sensitivity and isotropy of the displacement under applied forces and torques [16]. The displacement of a deformable part can be expressed based on Hooke’s Law, as shown in Equation (5).

(5) $$\begin{array}{c} \begin{array}{c} \delta_{6\times 1}={\bar{S}}_{6\times 6}\cdot {\bar{F}}_{6\times 1} \end{array} \end{array}$$

here, $\delta_{6\times 1}$ represents a displacement matrix and ${\bar{S}}_{6\times 6}$ is a compliance matrix, and ${\bar{F}}_{6\times 1}$ is a normalized force and torque matrix.

**Figure 4:**
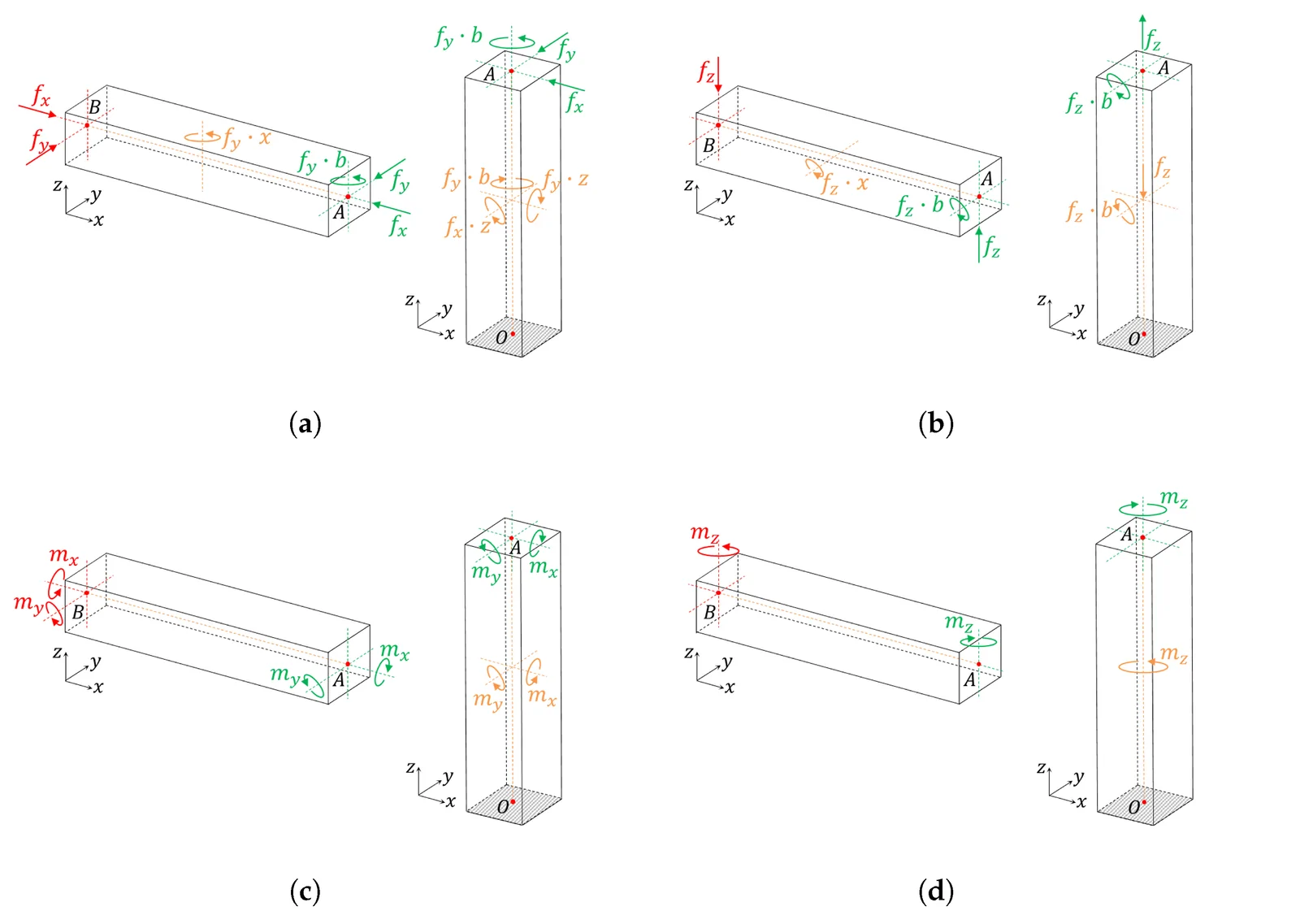
A Free-body diagram of the beam of a deformable part. Force and torque: (**a**) applied $F_{x}$ and $F_{y}$. (**b**) $F_{z}$. (**c**) applied $T_{x}$ and $T_{y}$. (**d**) applied $T_{z}$.

To calculate the displacement, we represent it as a free-body diagram of the beam for each axis, as shown in Figure 4a–d. Based on the principle of superposition, we divided it into two beams, the BA beam at node *B* and the AO beam at node *A*, and calculated them separately. We conducted a displacement analysis based on Castigliano’s beam theory to analyze the design parameters [21,22].

(6) $$\begin{array}{c} \begin{array}{c} \delta =\frac{\partial u}{\partial F}=\frac{\partial }{\partial F}\left(u_{F}+u_{T}\right) \end{array} \end{array}$$

(7) $$\begin{array}{c} \begin{array}{c} u_{F}=\int \frac{F^{2}}{2KE}\;,\;u_{T}=\int \frac{T^{2}}{2EI} \end{array} \end{array}$$

where $u_{F}$ represents the energy applied by force, $u_{T}$ represents the energy applied by torque, *K* is the area of the beams’ cross-section, *E* is the elastic modulus, and *I* is the moment of inertia. In the case of Figure 4a, the displacement along the *x*-axis and *y*-axis is equal because of its symmetric structure. This can be expressed in the following equation:

(8) $$\begin{array}{c} \begin{array}{c} \frac{\partial u_{BA}}{\partial F_{xy}}=\frac{\partial }{\partial F_{xy}}\int^{b}\frac{M_{z}^{2}}{2EI_{BA,z}}dx=\frac{b^{3}}{48E}\frac{sin^{2}\theta }{I_{BA,z}}F_{xy} \end{array} \end{array}$$

where $u_{BA}$ is the energy in the BA beam; $b=b_{2}+\frac{b_{1}}{2}$ is the length from node *B* to node *A*; $M_{z}=f_{y}x$, $f_{y}=\frac{sin\theta }{4}F_{xy}$, and $\theta =\frac{\pi }{4}$ are moments acting on the beam; and BA is the applied external force. The moment of inertia is $I_{BA,z}=\frac{h_{2}t^{3}}{12}$. Furthermore, the equation for beam AO, $\frac{\partial u_{AO}}{\partial F_{xy}}$ can be expressed as follows:

(9) $$\begin{array}{c} \begin{array}{c} \frac{\partial }{\partial F_{xy}}\int^{h}\left(\frac{1}{2E}\right)\left(\frac{M_{x}^{2}}{I_{AO,x}}+\frac{M_{y}^{2}}{I_{AO,y}}\right.+\left.\frac{1}{2}\frac{T_{z}^{2}}{GJ_{AO,z}}\right)dz\\ =\left\{\frac{h^{3}}{48E}\left(\frac{sin^{2}\theta }{I_{AO,x}}+\frac{cos^{2}\theta }{I_{AO,y}}\right)+\frac{b^{2}h}{16G}\left(\frac{sin^{2}\theta }{J_{AO,z}}\right)\right\}F_{xy} \end{array} \end{array}$$

where $h=h_{1}-\frac{b_{2}}{2}$ is the length from node *A* to node *O*, $M_{x}=f_{y}z$, $M_{y}=f_{x}z$, $f_{x}=\frac{cos\theta }{4}F_{xy}$, $T_{z}=f_{y}b$, *G* is the shear elastic modulus, the moments of inertia are $I_{AO,x}=\frac{b_{1}t^{3}}{12}$ and $I_{AO,y}=\frac{tb_{1}^{3}}{12}$, and the polar moment of inertia is $J_{AO,z}=\frac{b_{1}t}{12}\left(b_{1}^{2}+t^{2}\right)$. Also, in the case of Figure 4b, the displacement of the applied force $F_{z}$ of the beam BA and the beam AO can be expressed as follows:

(10) $$\begin{array}{c} \begin{array}{c} \frac{\partial u_{BA}}{\partial F_{z}}=\frac{b^{3}}{48E}\frac{1}{I_{BA,y}}F_{z} \end{array} \end{array}$$

(11) $$\begin{array}{c} \begin{array}{c} \frac{\partial u_{AO}}{\partial F_{z}}=\left(\frac{h}{16E}\frac{1}{K_{AO}}+\frac{h^{3}}{48E}\frac{1}{I_{AO,y}}\right)F_{z} \end{array} \end{array}$$

where $K_{AO}$ represents the area of the AO beam’s cross-section. Similarly, the equations for the cases applied by $T_{xy}$ in Figure 4c can be expressed as follows:

(12) $$\begin{array}{c} \begin{array}{c} \frac{\partial u_{AO}}{\partial T_{xy}}=\frac{h}{16E}\left(\frac{cos^{2}\theta }{I_{AO,x}}+\frac{sin^{2}\theta }{I_{AO,y}}\right)T_{xy} \end{array} \end{array}$$

In the case of Figure 4c, the energy in the BA beam applied $T_{xy}$ is $\frac{\partial u_{BA}}{\partial T_{xy}}=0$. And the equations for the cases applied $T_{z}$ in Figure 4d can be expressed as follows:

(13) $$\begin{array}{c} \begin{array}{c} \frac{\partial u_{AO}}{\partial T_{z}}=\frac{h}{16G}\frac{1}{J_{AO,z}}T_{z} \end{array} \end{array}$$

In the case of Figure 4d, the energy in the BA beam applied by $T_{z}$ is $\frac{\partial u_{BA}}{\partial T_{z}}=0$. Finally, through beam analysis, the compliance matrix is mathematically derived, allowing the displacement matrix to be expressed as follows:

(14) $$\begin{array}{c} \begin{array}{c} \delta_{6\times 1}={\left[\begin{array}{cccccc} ∆d_{s,x} & ∆d_{s,y} & ∆d_{n,z} & ∆r_{n,x} & ∆r_{n,y} & ∆r_{s,z} \end{array}\right]}^{T} \end{array} \end{array}$$

**Figure 5:**
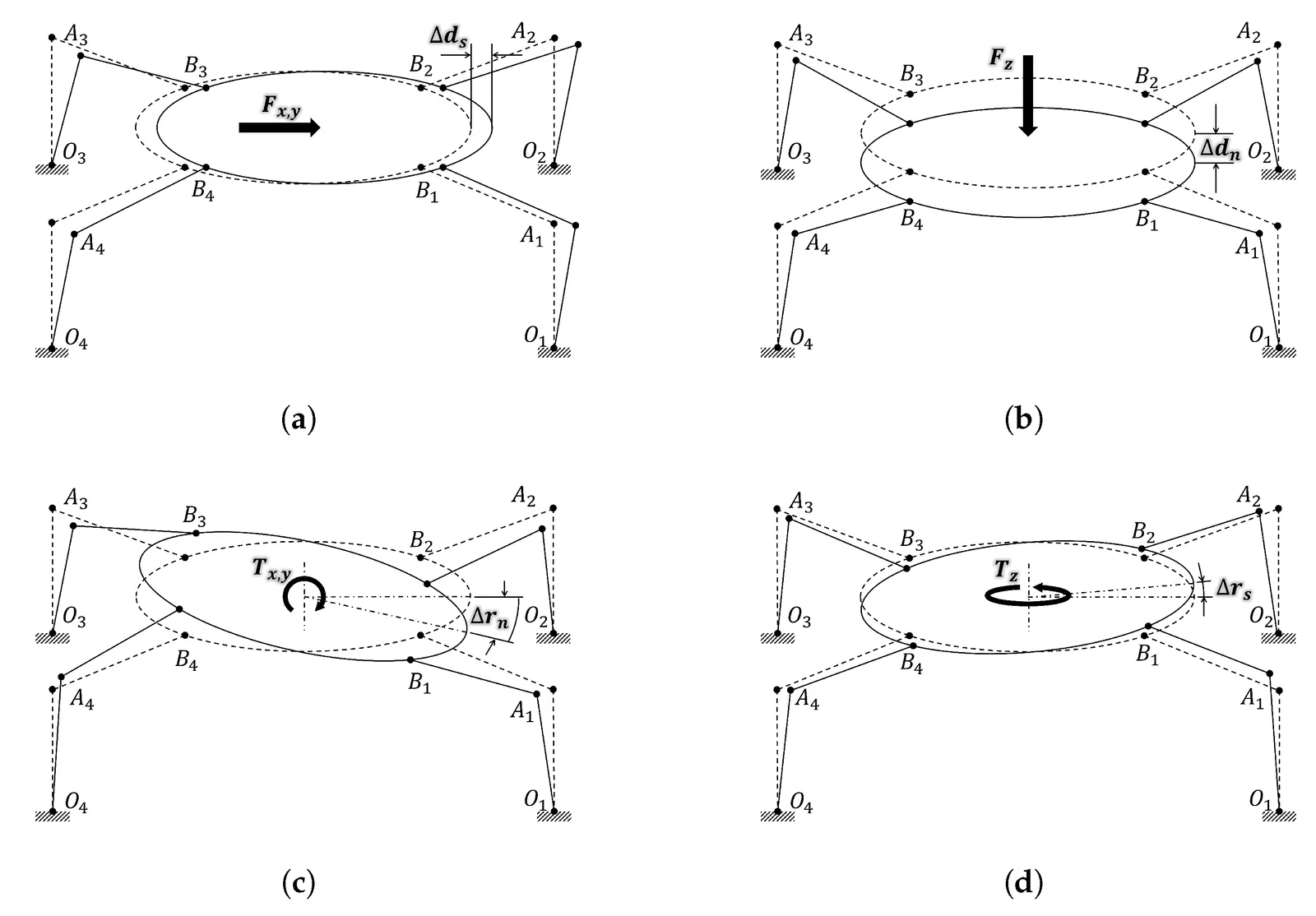
Mechanical models of the L-shaped deformable part and the displacement between applied loads: (**a**) $F_{x}$ and $F_{y}$. (**b**) $F_{z}$. (**c**) $T_{x}$ and $T_{y}$. (**d**) $T_{z}$.

The matrix δ in Equation (14) is composed of the displacement of each axis, as illustrated in Figure 5a–d. And the compliance matrix ${\bar{S}}_{6\times 6}$ can be expressed as a diagonal matrix composed of Equations (8)–(13) analyzed above [10,23,24].

(15) $$\begin{array}{c} \begin{array}{c} {\bar{S}}_{6\times 6}={\left[\begin{array}{ccc} S_{11} & \; & 0\\ \; & \ddots & \\ 0 & \; & S_{66} \end{array}\right]}^{T} \end{array} \end{array}$$

Due to structural symmetry along the *x*- and *y*-axes of a deformable part, $S_{11}$ and $S_{22}$ are the same.

(16) $$\begin{array}{c} \begin{array}{cc} S_{11,22} & =\left\{\frac{b^{3}}{48E}\frac{sin^{2}\theta }{I_{BA,z}}+\frac{h^{3}}{48E}\left(\frac{sin^{2}\theta }{I_{AO,x}}+\frac{cos^{2}\theta }{I_{AO,y}}\right)\right.\\ & +\left.\frac{b^{2}h}{16G}\left(\frac{sin^{2}\theta }{J_{AO,z}}\right)\right\}F_{x,y,max} \end{array} \end{array}$$

(17) $$\begin{array}{c} \begin{array}{c} S_{33}=\left(\frac{b^{3}}{48E}\frac{1}{I_{BA,y}}+\frac{h}{16E}\frac{1}{K_{AO}}+\frac{h^{3}}{48E}\frac{1}{I_{AO,y}}\right)F_{z,max} \end{array} \end{array}$$

Similarly, $S_{44}$ and $S_{55}$ also have the same expression due to their symmetric structure.

(18) $$\begin{array}{c} \begin{array}{c} S_{44,55}=\frac{h}{16E}\left(\frac{cos^{2}\theta }{I_{AO,x}}+\frac{sin^{2}\theta }{I_{AO,y}}\right)T_{x,y,max} \end{array} \end{array}$$

(19) $$\begin{array}{c} \begin{array}{c} S_{66}=\frac{h}{16G}\frac{1}{J_{AO,z}}T_{z,max} \end{array} \end{array}$$

The normalization of forces and torques is necessary to evaluate their effect on displacements using the compliance matrix. Because forces and torques have different units and magnitudes, converting them into dimensionless quantities is essential for effective evaluation and comparison. The matrix ${\bar{F}}_{6\times 6}$ is composed of normalized forces and torques and is expressed as follows:

(20) $$\begin{array}{c} \begin{array}{c} {\bar{F}}_{6\times 1}={\left[\begin{array}{cccccc} \frac{F_{x}}{F_{x,max}} & \frac{F_{y}}{F_{y,max}} & \frac{F_{z}}{F_{z,max}} & \frac{T_{x}}{T_{x,max}} & \frac{T_{y}}{T_{y,max}} & \frac{T_{z}}{T_{z,max}} \end{array}\right]}^{T} \end{array} \end{array}$$

## 3. Isotropic Optimization

### 3.1. Deformable Part with Optimal Design

The compliance matrix is quantified, and optimizing the design parameters for the initial design criteria for the deformable part is necessary. According to Uchiyama et al., as a method for isotropic optimal design, the condition number of the normalized compliance matrix is a crucial design index [23,24]. Based on the condition number of the normalized compliance matrix $\bar{S}$ obtained by the analysis earlier, the objective function can be expressed as follows:

(21) $$\begin{array}{c} \begin{array}{c} f\left(x\right)=Cond(\bar{S}\left(b_{1},b_{2},h_{1},h_{2},t\right)) \end{array} \end{array}$$

As in Equation (21), the design parameters $b_{1},b_{2},h_{1},h_{2},t$ at the point where the objective function is minimized serve as a criterion for the optimal design of the deformable part. Optimization is carried out to find the five optimal design parameters by defining the objective function and constraints as follows:

(22) $$\begin{array}{c} \begin{array}{c} \mathrm{minimize}\;f=Cond(\bar{S}) \end{array} \end{array}$$

(23) $$\begin{array}{c} \begin{array}{cc} \mathrm{subject}\;\mathrm{to}\; & \delta_{min}\leq S_{ii}\leq \delta_{max}\;\left(i=1,\cdots ,6\right)\\ & \sigma_{max}\leq \sigma_{Y}\\ & 2b_{1}-b_{2}\leq 0\\ & 2h_{2}-h_{1}\leq 0\\ & t-b_{2}\leq 0\\ & t-h_{1}\leq 0 \end{array} \end{array}$$

where $\delta_{min}$ and $\delta_{max}$ represent the minimum and maximum displacements, respectively, and $\sigma_{max}$ represents the maximum stress in the deformable part. Also, the material is AL7075-T6, and $\sigma_{Y}$ denotes the yield strength. To find the optimal design parameters $b_{1}\;b_{2}\;h_{1}\;h_{2}\;t$, the sequential quadratic programming (SQP) optimization solver is used. The results of these calculations are presented in Table 1.

Using the optimal parameters from Table 1, a compliance matrix $\bar{S}$ can be derived, as shown in Equation (24).

(24) $$\begin{array}{c} \begin{array}{c} {\bar{S}}_{6\times 6}={\left[\begin{array}{cccccc} 0.0501 & 0 & 0 & 0 & 0 & 0\\ 0 & 0.0501 & 0 & 0 & 0 & 0\\ 0 & 0 & 0.0554 & 0 & 0 & 0\\ 0 & 0 & 0 & 0.0511 & 0 & 0\\ 0 & 0 & 0 & 0 & 0.0511 & 0\\ 0 & 0 & 0 & 0 & 0 & 0.0631 \end{array}\right]}^{T} \end{array} \end{array}$$

As shown in Equation (20), since matrix $\bar{F}$ is dimensionless, the elements of the compliance matrix $\bar{S}$ ultimately represent the displacement δ in each axis direction.

### 3.2. Sensing Electrode Design

After optimizing the design parameters, the electrode’s area and initial distance also need to be determined. The arrangement of the sensing electrodes affects the performance of the capacitive sensor. As shown in Equation (4), ∆C is affected not only by the displacement ∆d, but also by the electrode area *A* and the initial gap $d_{init}$.

**Figure 6:**
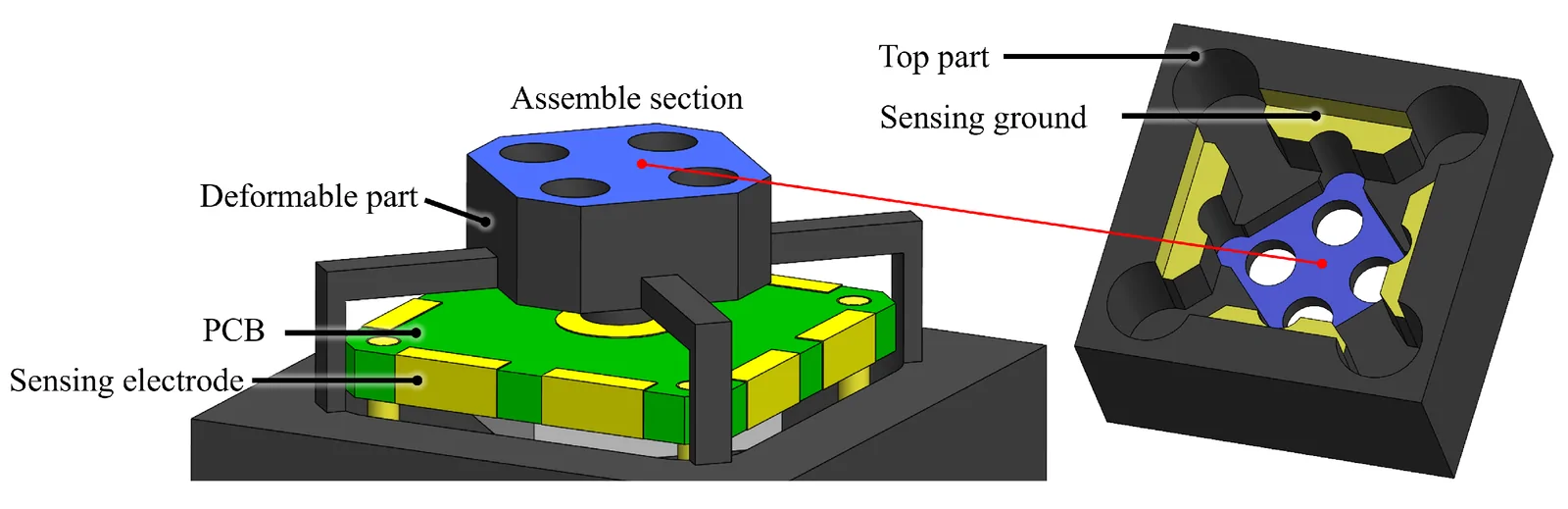
Arrangement of capacitive sensing electrodes and composition of sensing unit.

As shown in Figure 6, the capacitive sensing unit should be positioned close to the deformable part to measure its displacement accurately. Also, the sensing unit should be configured to facilitate the arrangement of the GND area and sensing electrodes. The top part, which serves the role of transmitting external force/torque load, is designed with an internal sensing ground area. When assembled with the deformable part, it is configured to form the capacitive sensing unit automatically. The sensing electrode area *A* on the arranged PCB and the initial air gap $d_{init}$ between the electrode and the ground should be determined. If the area and initial air gap are arbitrarily selected without criteria, the deformable part designed with isotropy may fail in its intended performance. Therefore, when designing capacitive force/torque sensors, isotropy should be considered not only for the deformable part, but also when selecting the electrode area and initial air gap. Research on capacitive force/torque sensor design focuses primarily on analyzing the deformable part. The selection of electrode area and air gap criterion parameters is also a critical issue in sensor design. This section introduces the method for selecting the electrode area and initial gap. According to Equation (4), the capacitance, which depends on three variables, displacement, electrode area, and initial air gap, can now be expressed as a function of two variables: electrode area and initial air gap.

**Figure 7:**
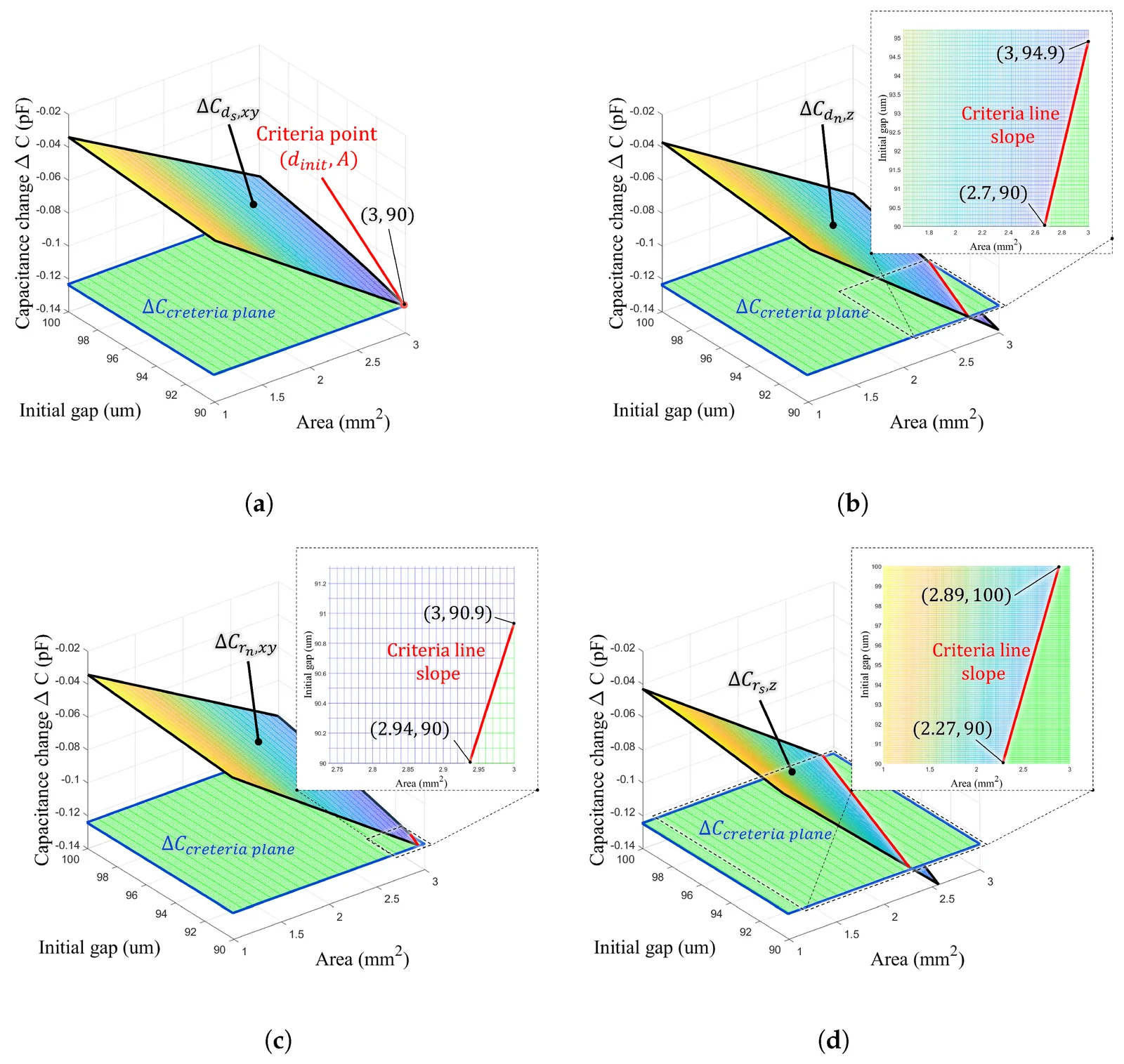
Capacitance change as a function of area and initial gap, and capacitance change loaded by each axis force and torque. (**a**) $∆C_{d_{s},xy}$ by $F_{xy}$ (**b**) $∆C_{d_{n},z}$ by $F_{z}$ (**c**) $∆C_{r_{n},xy}$ by $T_{xy}$ (**d**) $∆C_{r_{n},z}$ by $T_{z}$.

As shown in Figure 7, the change in capacitance can be represented as a three-dimensional function of electrode area and initial gap. Figure 7a–d illustrates the capacitance change caused by force and torque along each axis. Identifying the criteria point is necessary to select the electrode area and initial gap while considering isotropy. As shown in Figure 7a, the point with the smallest capacitance change caused by $F_{xy}$ is selected as the criteria point, and the criteria plane is obtained based on the criteria point. The criteria plane becomes a constant; this approach allows the identification of overlapping lines between the capacitance change and the criteria plane.

As shown in Figure 7b–d, the linear equation of the red line (criteria line) can be derived. This linear equation can be used as a criterion for selecting the electrode area and initial gap, enabling the design of the sensing electrode with consideration for isotropy.

(25) $$\begin{array}{c} \begin{array}{cc} \; & d_{init,Fxy}=90\;\mu m,\;A_{Fxy}=3\;{\mathrm{mm}}^{2}\\ \; & d_{init,Fz}=15.3A_{Fz}+48.9\\ \; & d_{init,Txy}=1.5A_{Txy}+86.4\\ \; & d_{init,Tz}=16.1A_{Fz}+44.4 \end{array} \end{array}$$

The linear equation can be expressed as Equation (25), and the sensing electrode design responsive to $F_{xy}$ can be determined with a 90 µm initial gap and a 3 ${\mathrm{mm}}^{2}$ area. The sensing electrodes responsive to $F_{z}$, $T_{xy}$, and $T_{z}$ can be designed by adjusting the initial gap and area according to the linear equation.

As defined in Equation (4), the capacitance change can be expressed as a function of three parameters: electrode area, initial gap, and displacement. Through Equation (5)–(19), the displacement parameters for each axis are obtained. The displacement parameter is summarized and expressed as the compliance matrix $\bar{S}$, as shown in Equation (24). Hence, the capacitance change is determined by the two design parameters of the sensing electrode: the area and the initial gap. Finally, the area *A* and initial gap $d_{init}$ can be selected based on Equation (24) to design the sensing electrode.

**Figure 8:**
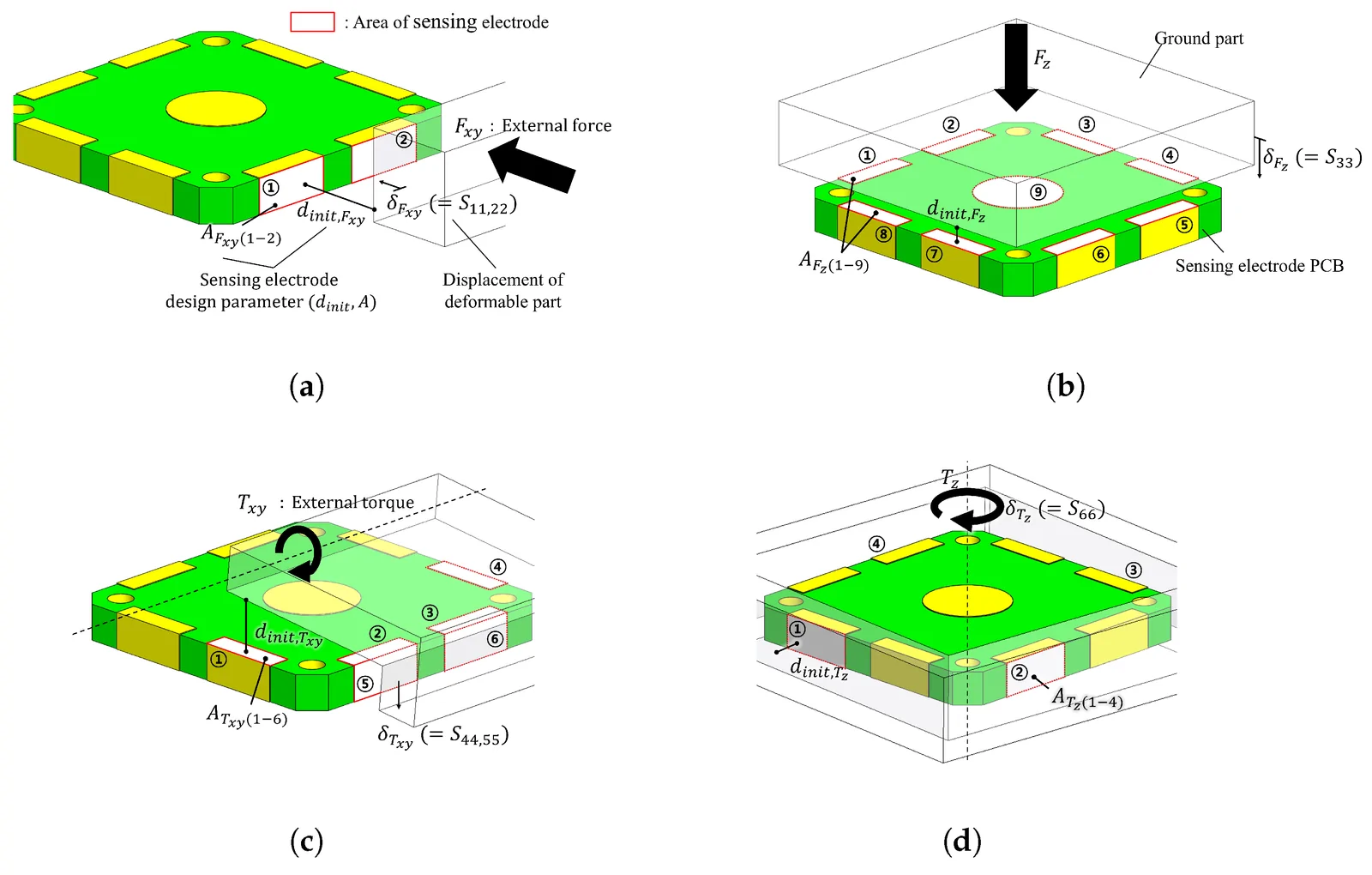
The selection of the initial gap and area of the sensing electrode, and the response region of the sensing electrode under applied force and torque: (**a**) $F_{xy}$, (**b**) $F_{z}$, (**c**) $T_{xy}$, (**d**) $T_{z}$.

As shown in Figure 8, the sensing electrode PCB consists of nine sensing cells. The white-marked area represents the sensing electrode areas, and the numbers 1–9 indicate the quantity of sensing electrode areas. Figure 8a illustrates the case when $F_{xy}$ is applied, the displacement is represented as $S_{11,22}$, and two sensing cells respond to this displacement. Since the $F_{xy}$ application case is set as the criteria point, the electrode area is set to 3 ${\mathrm{mm}}^{2}$ and the initial gap to 90 µm. Figure 8b illustrates the case when $F_{xy}$ is applied. The displacement is $S_{33}$, and nine sensing cells respond. From a practical perspective, if the initial gap is set to 90 µm in the case of $F_{z}$, the same value as in the $F_{xy}$ case, the area of the nine sensing cells can be determined accordingly. Figure 8c illustrates the case when $T_{xy}$ is applied. The displacement is represented as $S_{44,55}$, and six sensing cells respond to this displacement. As shown in Figure 8a,b, the initial gap responding to $F_{xyz}$ is set to 90 µm. Accordingly, the areas of the six sensing cells responding to $T_{xy}$, as shown in Figure 8c, are automatically determined. Figure 8d illustrates the case when $T_{z}$ is applied. The displacement is $S_{66}$, and four sensing cells respond to this displacement. Since the initial gap and area have already been determined in the previous cases, $F_{xyz}$ and $T_{xy}$, the area and initial gap responding to $T_{z}$ are also automatically determined. The displacement parameter is determined by analyzing the deformable part, as shown in Equation (5)–(19). Based on Equation (25), the function of capacitance change is derived with the electrode area and the initial gap as variables. This design method for selecting the design parameters, such as the area and initial gap, is outlined, and the sensing electrode PCB is subsequently designed.

## 4. Development of Miniature Six-Axis F/T Sensor

### 4.1. Fabrication and Specification

**Figure 9:**
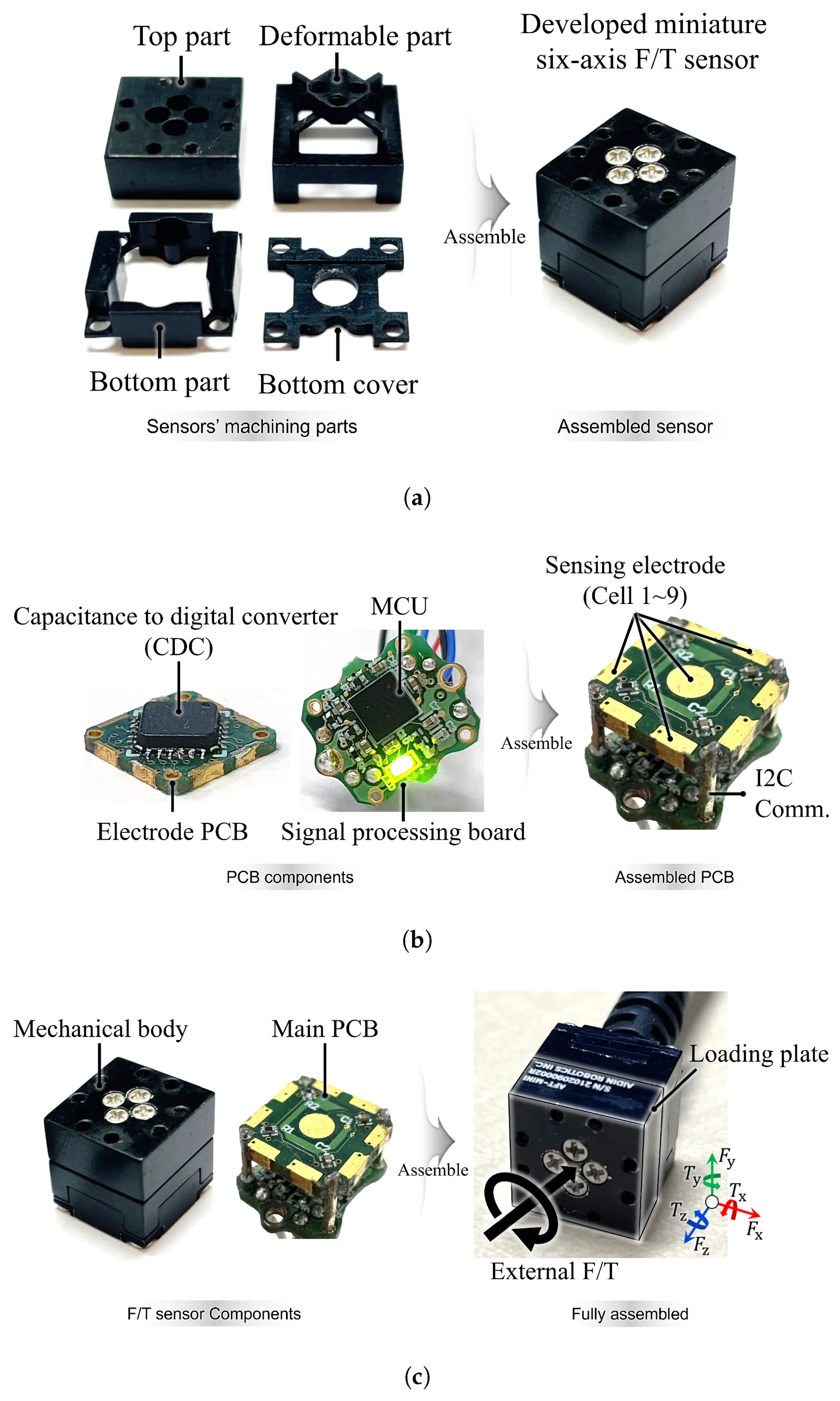
Developed sensor and components. (**a**) Mechanical parts (top part, deformable part, bottom part, bottom cover. (**b**) PCB components (sensing electrode, signal processing board). (**c**) Miniature F/T sensor.

The design parameters for the deformable part, which consider isotropy, the electrode area, and the initial gap between sensing electrodes, are determined. Based on this, a compact miniature six-axis force/torque sensor is developed and introduced in this section. As shown in Figure 9a,b, the mechanical components of the sensor are composed of four parts: the top part, deformable part, bottom part, and bottom cover. Additionally, the PCB consists of two parts: the sensing electrode PCB and the signal processing board.

The sensing electrode and signal processing board are integrated into the sensor, eliminating the need for additional devices. The deformable part is designed with an easily machinable structure, facilitating mass production and cost-effective manufacturing [16,25]. The sensing electrode includes a capacitance digital converter (CDC) chip (AD7147, Analog Devices) that converts capacitance into digital signals and is connected to the signal processing board via $I^{2}C$ communication. The ARM processors (STM32F042, ST) on the signal processing PCB collect data and output it through CAN communication.

The assembly is relatively easy due to the small number of parts and a simple bolt-on assembly process. The specifications of the developed sensor are shown in Table 2. It is a cube-shaped design with a length of 10 mm, a force/torque sensing range of 15 N and 75 N-mm, CAN communication, and a sampling rate of 100 Hz.

### 4.2. Calibration Method

**Figure 10:**
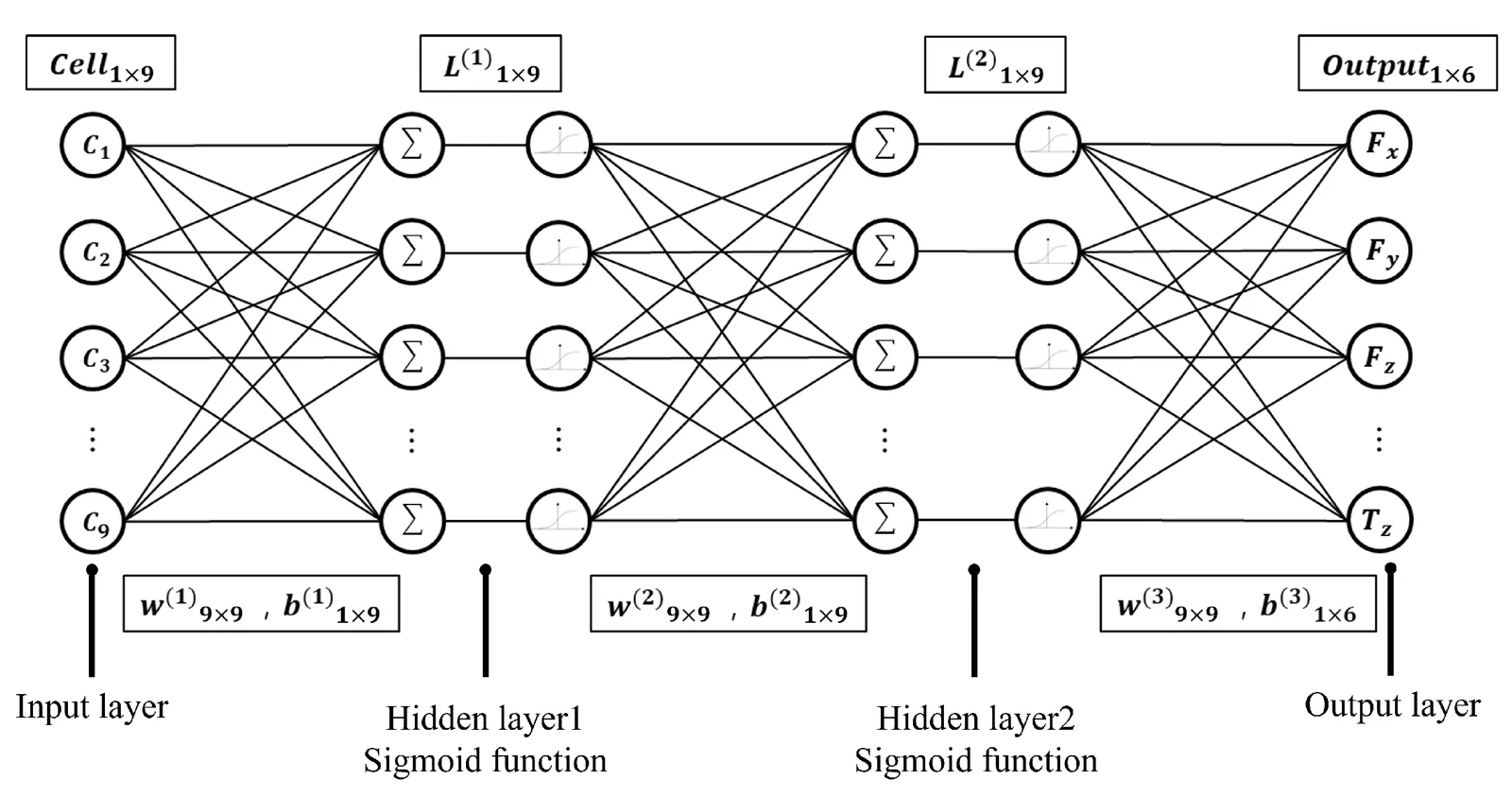
Model of the ANN calibration method.

The calibration method is one of the critical issues that determine the performance of the force/torque sensor during the development process [26]. In particular, capacitive force/torque sensors’ nonlinearity characteristic is often a challenging issue during the calibration process. Typically, capacitive sensors use calibration methods such as linear interpolation and second-order nonlinear fitting techniques; however, these methods have limitations in effectively addressing capacitance’s nonlinearity. The nonlinearity of capacitance can be efficiently linearly fitted using the sigmoid function, the activation function in neural networks. For this reason, neural network-based calibration methods have been widely used to calibrate capacitive force/torque sensors and address nonlinearity [27]. The developed miniature six-axis F/T sensor has been applied with a neural network-based calibration method. The calibration model is structured as shown in Figure 10. The input data consists of the capacitance measured from nine sensing electrodes, which are normalized and used as inputs to the network. The output data corresponds to the six-axis force/torque that caused the capacitance change. These values are also normalized and used to train the network. The model includes two hidden layers, each with weights of size 9×9 and biases of size 1×9. The activation function used is the sigmoid function. The training model utilized the Adam optimizer, and the network is trained using the mean square error as the loss function. The optimal weights and bias parameters obtained through neural network training are used as the sensor’s calibration matrix.

### 4.3. Calibration and Experiment

**Figure 11:**
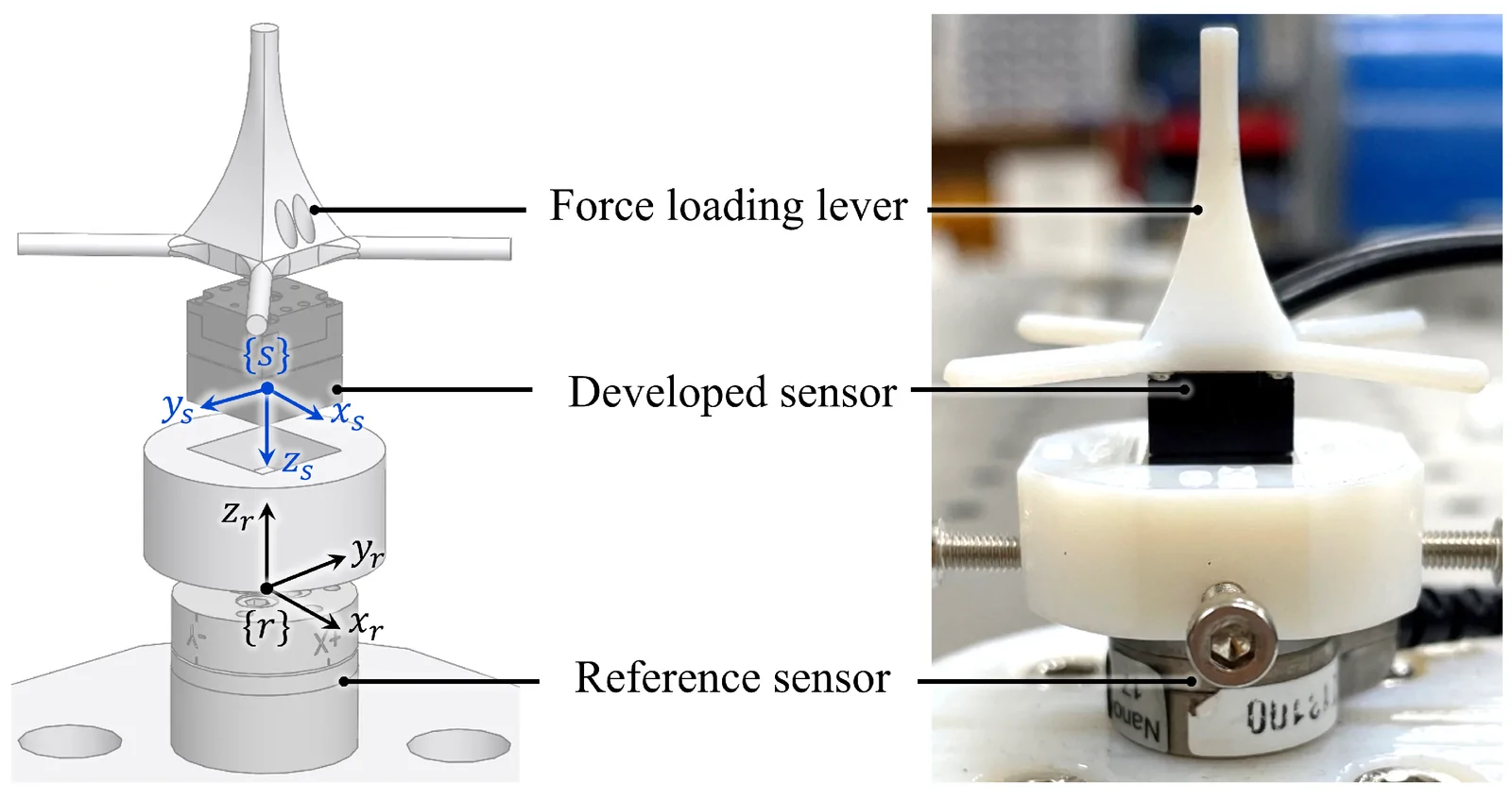
Experimental setup for sensor calibration.

**Figure 12:**
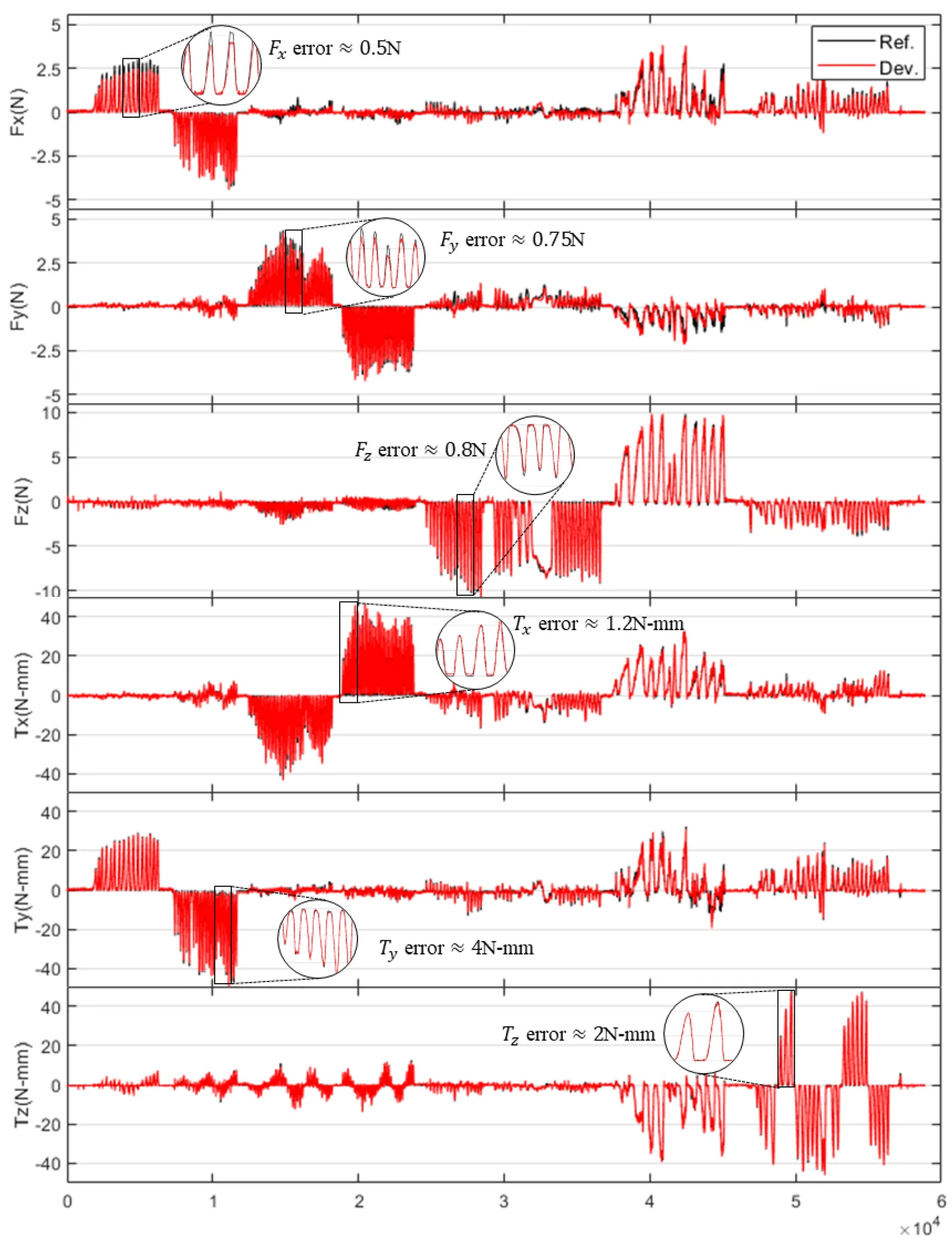
Comparison of evaluated results with developed F/T sensor and reference F/T sensor.

The calibration experimental setup as illustrated in Figure 11. The experimental setup consists of a force loading lever, the developed six-axis F/T sensor, connection jigs, and a reference six-axis F/T sensor (Nano-17, ATI). The training dataset is collected manually, ensuring that only the force $F_{z}$ and the torque $T_{z}$ are loaded in a single-axis direction. We collect data on $F_{x}$, $T_{y}$ and $F_{y}$, $T_{x}$ where coupling occurs between force and torque.We also collect random force and torque data from all axes.As shown in Figure 12, a comparative evaluation is conducted between the reference sensor and the developed sensor. Ideally, the evaluation data should be represented by a single force or torque for each axis [28,29]. However, due to the manual force torque loading process, it can be observed that the forces and torques are coupled. Therefore, to determine the feasibility of the developed sensor, the performance should be evaluated by calculating the error between the reference sensor and the developed sensor. The accuracy and linearity are analyzed based on the full-scale output error of force and torque, calculated within ranges of 15 N and 75 N-mm, respectively. Additionally, the resolution is analyzed using the standard deviation from the collected data. The developed sensor’s accuracy, linearity, and repeatability are evaluated via an analysis; the results are presented in Table 3. Accuracy and linearity are calculated based on the full-scale output (FSO) of the sensor, and resolution is calculated as the standard deviation of the data.

**Figure 13:**
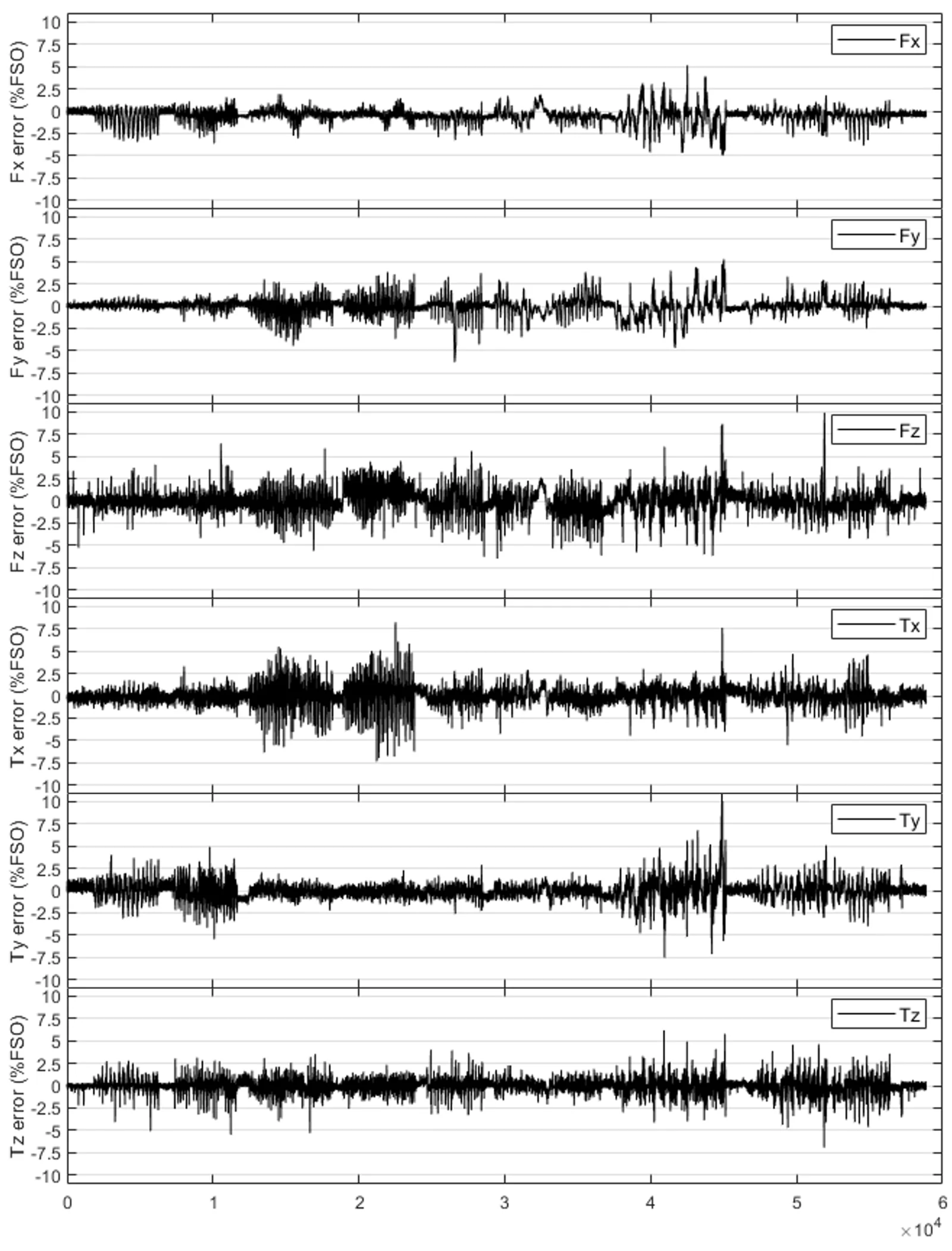
The error results between the reference sensor and the developed sensor.

The relative error results are presented in Figure 13. The mean error values of the measured forces are 0.16, 0.18, and 0.24. The mean error values of the measured torques are 0.88, 0.90, and 0.67. In the case of maximum force error, the values are 4.5, 6.3, and 9.9, and the maximum torque error values are 8.2, 10.2, and 6.9. The relative errors calculated from the mean square error results are presented in Table 4.

## 5. Conclusions

This study developed a miniature capacitive six-axis force/torque (F/T) sensor optimized for isotropy and compact design. By optimizing the compliance matrix, a condition number of 1.261 is achieved, ensuring uniform sensitivity across all six axes. Additionally, a systematic method for designing sensing electrodes, including the selection of electrode area and initial gap, is established using linear equations derived from capacitance change analysis. This approach preserves isotropy and enhances measurement accuracy. Experimental validation confirmed the sensor’s performance, with accuracies between 98.23% and 99.53%, minimal relative errors, and a high resolution. Neural network-based calibration effectively addressed nonlinearity, ensuring reliable measurements.

**Figure 14:**
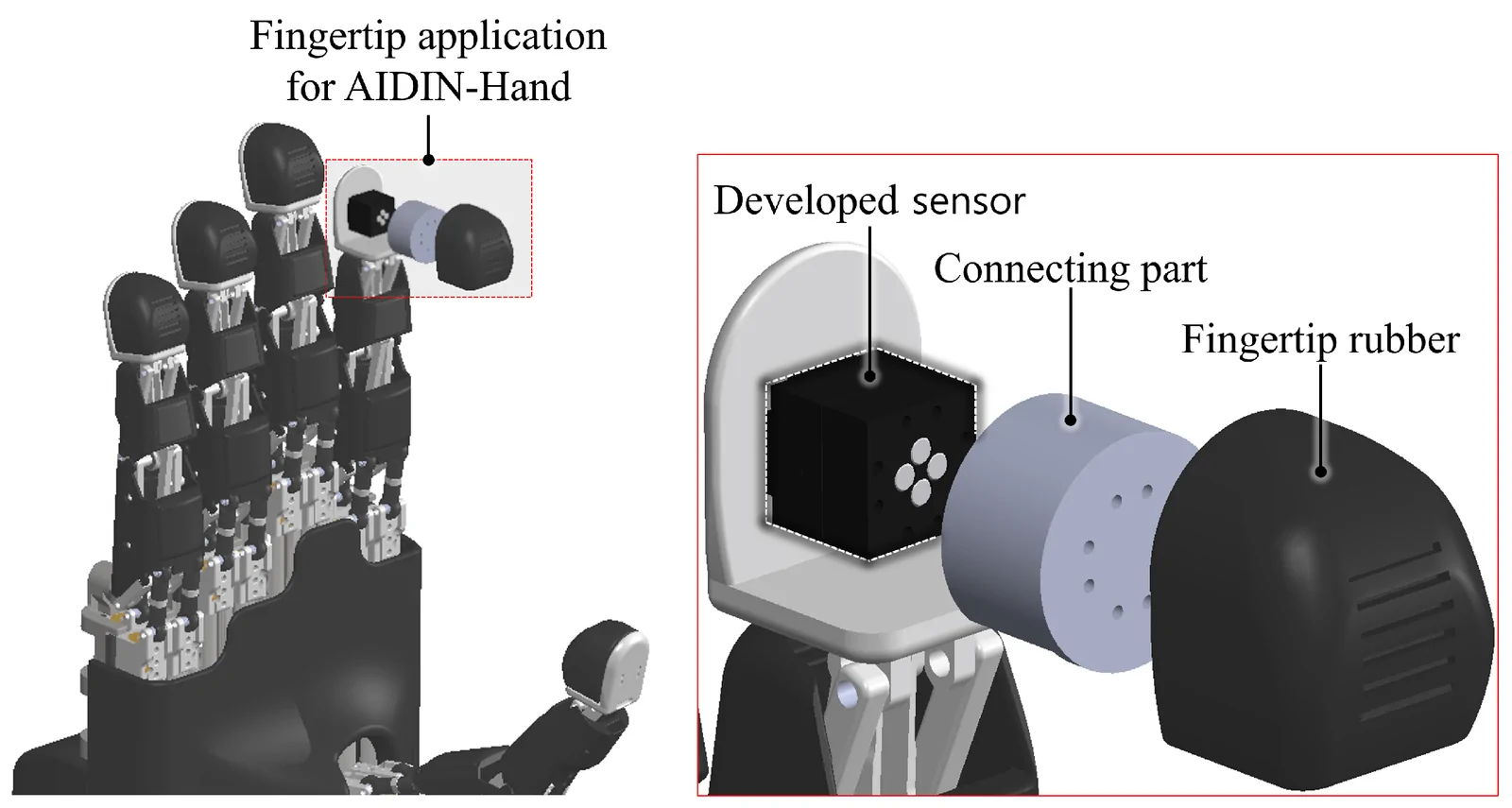
An application example of miniature six-axis force/torque sensors for robot hand (also known as the AIDIN-Hand).

In conclusion, the sensor combines compactness and isotropy, making it suitable for advanced robotic applications requiring precise force control. The design framework provides a foundation for future developments in miniature F/T sensors. Furthermore, the sensor’s compact size (10 × 10× 10 mm) and integration-friendly design make it particularly suited for embedding into robotic fingertips, as shown in Figure 14. The developed sensor addresses issues such as limited space and the need for lightweight components, paving the way for advanced fingertip applications in the advanced robotics field.

## Figures and Tables

**Table 1:** Results of optimized design parameters.

Condition Number	$b_{1}$	$b_{2}$	$h_{1}$	$h_{2}$	*t*	Unit
1.261	0.49	1.15	3.45	0.37	0.65	mm

**Table 2:** Specifications of the developed six-axis F/T sensor.

Property	Value	Unit
Dimension	10 × 10 × 10	mm
Force	15	N
Torque	75	N-mm
Communication	CAN	
Sampling rate	100	Hz

**Table 3:** The specifications of the developed six-axis F/T sensor.

	Accuracy	Linearity	Resolution
$F_{x}$	98.97	98.42	0.74
$F_{y}$	98.87	98.23	0.77
$F_{z}$	98.23	97.59	0.39
$T_{x}$	99.37	99.12	0.52
$T_{y}$	99.38	99.09	0.34
$T_{z}$	99.53	99.33	0.22

**Table 4:** The error results of the developed sensor.

Quantity	Value	unit
Relative error of forces	0.16, 0.18, 0.24	%FSO
Relative error of torques	0.88, 0.90, 0.67	%FSO

## Data Availability

The original contributions presented in this study are included in the article. Further inquiries can be directed to the corresponding author.
